# Social Disparities in Dental Insurance and Annual Dental Visits Among Medically Insured Patients With Diabetes: the Diabetes Study of Northern California (DISTANCE) Survey

**Published:** 2010-04-15

**Authors:** Howard H. Moffet, Dean Schillinger, Jane A. Weintraub, Nancy Adler, Jennifer Y Liu, Joe V. Selby, Andrew J Karter

**Affiliations:** Kaiser Permanente, Division of Research; California Department of Public Health, Sacramento, California, and University of California, San Francisco, School of Medicine, San Francisco General Hospital, San Francisco, California; University of California, San Francisco, School of Dentistry, San Francisco, California; University of California, San Francisco, Departments of Psychiatry and Pediatrics and Center for Health and Community, San Francisco, California; Kaiser Permanente, Division of Research, Oakland, California; Kaiser Permanente, Division of Research, Oakland, California; University of Washington, Department of Epidemiology, School of Public Health and Community Medicine, Seattle, Washington, and Kaiser Permanente, Division of Research, Oakland, California

## Abstract

**Introduction:**

People with diabetes are at increased risk of periodontal disease and tooth loss. *Healthy People 2010* set a goal that 71% or more of people with diabetes should have an annual dental exam.

**Methods:**

We assessed dental insurance and annual dental visits among dentate respondents from the Diabetes Study of Northern California (DISTANCE) Survey cohort (N = 20,188), an ethnically stratified, random sample of patients with diabetes aged 30 to 75 years receiving medical care from Kaiser Permanente Northern California. We calculated predicted probabilities for an annual dental visit (PPADV) by using regression models that incorporated age, sex, education level, annual household income, and self-reported race/ethnicity, stratified by whether the respondent had dental insurance.

**Results:**

Among 12,405 dentate patients, 9,257 (75%) had dental insurance. Annual dental visits were reported by 7,557 (82%) patients with dental insurance and 1,935 (61%) patients without dental insurance. The age-sex adjusted odds ratio for an annual dental visit was 2.66 (95% confidence interval, 2.33-3.03) for patients with dental insurance compared to those without dental insurance. For patients with dental insurance, the PPADV was 71% or more for all except those with the lowest household income. In contrast, for those without dental insurance, the PPADV was less than 71% for all except those with the most education or the highest income. We found some racial/ethnic subgroups were more likely than others to take advantage of dental insurance to have an annual dental visit.

**Conclusions:**

Patients with diabetes in this managed care population who lacked dental insurance failed to meet the *Healthy People 2010 *goal for an annual dental visit. An increased effort should be made to promote oral health among people with diabetes.

## Introduction

People with diabetes are at increased risk of periodontitis and tooth loss (1,2); common inflammatory pathways (3) appear to be associated with hyperglycemia, periodontitis, and diabetes complications (4). The consequences of periodontitis and tooth loss include impaired chewing ability and poorer oral health-related quality of life and general health (5). Thus, good oral health may contribute to good management of diabetes. People with tooth loss may also experience dissatisfaction with appearance, avoid social contacts, have trouble speaking, be more likely to be obese, eat less fruits and vegetables, and have lower biochemical levels of some nutrients and dietary fiber (6).

As many as 1 in 3 people in the United States lacks dental insurance (7), but the numbers are higher among poor, low-income, or retired people (8) and the medically uninsured (9). Data from the 1999 National Health Interview Survey revealed that 71% of dentate adults aged 55 years or older in the United States made at least 1 dental visit in the preceding year, but rates were lower among those without dental insurance (68%), racial/ethnic minorities (eg, Hispanics, 55%; non-Hispanic blacks, 49%), and the poor (43% for people below the federal poverty level) (10). Beyond dental insurance, differences in receipt of dental care may be attributable to willingness or ability to pay for care, patient preferences or beliefs about dental care (10), lack of transportation, inflexible employment, and individual functional limitations or disability (7). Sex and race differences in seeing a dentist have been observed, even among those with insurance (8). Those who avoid seeing a dentist except when necessary tend to have poorer oral health (11). Dentate people with diabetes are less likely than dentate people without diabetes to visit a dentist (12). Finally, among people with diabetes, larger disparities have been observed in dental care visits by sex, race/ethnicity, socioeconomic status, and education level than for use of other health care services (2,13).

An annual dental visit, typically for a routine checkup or cleaning, represents the minimum use of professional dental care. *Healthy People 2010* (HP 2010) reports that 56% of people with diabetes aged 2 years or older had at least 1 annual dental visit in 1997 and set a goal that 71% or more of people with diabetes have at least 1 dental examination annually (71% being the prevalence among all dentate adults [10]) (14). To date, no studies have adequately examined the role of dental insurance in social disparities in annual dental visits among medically insured people with diabetes. We sought to assess whether the HP 2010 goal was being met in a medically insured population. We hypothesized that having dental insurance facilitates seeing a dentist and that social disparities (by race/ethnicity, education level, or annual income) may interfere with achieving this goal.

## Methods

This study is from the Diabetes Study of Northern California (DISTANCE) Survey, whose aim is to identify potentially modifiable factors that explain the pathways by which social factors, particularly educational attainment and race/ethnicity, are related to diabetes-related health outcomes. The DISTANCE Survey cohort were respondents to a telephone interview or self-administered, 184-item questionnaire (online, written, or short [40-item] written versions were offered) conducted in 2005 and 2006 among an ethnically stratified (African American, Asian, white, Latino, and unknown ethnicity), random sample of 40,735 members with diabetes (with oversampling of nonwhite, minority patients), aged 30 to 75 years receiving medical care from Kaiser Permanente Northern California. A full description of the survey and cohort has been published (15); general information and the complete survey are available at http://distancesurvey.org.

The Kaiser Permanente membership includes employed and retired people and their families; it closely approximates the catchment area population by race/ethnicity and socioeconomic status except for the extremes of the income distribution (16). Health plan membership does not include dental care.

Our main outcome of interest in this study was an annual dental visit. The DISTANCE Survey included validated questions about receipt of preventive dental care during the past 3 years ("seeing a dentist for routine checkups or cleanings") with visit frequency collapsed dichotomously to "annual" (ie, at least once a year) or "less than annual" (17). Additional questions were about current dental insurance, current flossing habits, and tooth loss (18). The oral health items used in the questionnaire are in the Appendix; the short version of the questionnaire did not include the questions about dental insurance, flossing, or tooth loss. All data (age, sex, race/ethnicity, employment status, education, income) were obtained from the questionnaire, except hemoglobin A1c (HbA1c) values, which were obtained from electronic health records.

For this study, we excluded patients who had no teeth because they may not recognize a need for dental visits, they are not at risk for periodontal disease or further tooth loss, and they have markedly lower rates of dental visits (10). We calculated prevalence of dental insurance by race/ethnicity and employment status among patients who answered the question about dental insurance. Among patients who also answered the question about receipt of preventive dental care, we calculated frequency distributions of patient characteristics overall and dichotomized by annual or less than annual dental visits; χ^2^ tests were used to determine significant differences.

To explore the predictors of dental care, we specified multivariate logistic models that regressed annual dental visits on age, sex, annual income, education level, race/ethnicity, and having dental insurance by using the SAS survey logistic procedure (SAS Institute, Cary, North Carolina) to account for nonproportional sampling fractions in the race-stratified sample. We specified additional models with cross-product terms to evaluate significant interactions between our exposures of interest and hypothesized effect modifiers. We used the logistic model to derive predicted probabilities for annual dental visits (PPADV), since the outcome was not rare and, thus, odds ratios (ORs) would be unsuitable estimates of effect size (19). This research was approved by the institutional review boards of the Kaiser Foundation Research Institute and the University of California, San Francisco.

## Results

The questionnaire was completed by 20,188 people (10,429 telephone interviews, 4,288 written surveys, 2,393 short written surveys, and 3,078 online surveys). We used an algorithm endorsed by the Council of American Survey Research Organizations (20) to determine a response rate of 62% (we assumed people unable to be contacted had the same rate of eligibility as those who were contacted and, therefore, removed an estimated number of ineligible nonrespondents from the denominator). Few baseline variables differed between respondents and nonrespondents, and analyses of associations between race/ethnicity or education level and poor glycemic control (HbA1c >7%) detected no response bias (for survey response interaction with race/ethnicity, *P* = .55, and with education, *P* = .28) (15). We excluded 1,619 people who indicated they had no teeth or wore full dentures, leaving 18,569 dentate people with diabetes.

Among the 18,569 dentate adults with diabetes in the DISTANCE cohort, 12,405 eligible people answered both the dental insurance question and the dental visit question; the main analyses were based on this sample (Table 1). Data for the dental insurance question were missing for 6,164 people: 3,639 were from telephone interviews, usually because the interview had been discontinued before that question; 2,142 were from the short version of the survey that did not include the dental insurance question; 207 were from online surveys; and 176 were from written surveys.

In this sample, 75% (n = 9,257) of patients had dental insurance. Prevalence of dental insurance varied by race/ethnicity (African Americans, 82%; Latinos, 74%; whites, 68%; Chinese, 73%; Filipinos, 83%; other/mixed race, 74%) and by employment status (employed, 85%; retired, 60%; disabled, 63%; other [eg, student, homemaker], 69%).

Annual dental visits were reported by 77% of patients, 82% with dental insurance and 61% without dental insurance. Overall, no dental visits in the past 3 years were reported by 5%, and this proportion differed between those with dental insurance (3%) and those without (9%).

We observed modest social disparities in dental visits by race/ethnicity (annual visits reported by 73% of African Americans and Latinos vs 83% of Chinese and Filipinos). We observed larger differences by socioeconomic status: annual visits were reported by 66% of patients without a high school diploma compared with 86% of college graduates, and 59% of patients with annual household incomes of $15,000 or less, compared with 85% of those with incomes of $65,000 or more. Health behaviors and self-rated health and psychosocial factors were also associated with dental visits. Annual dental visit rates of less than 71% were observed among those who floss occasionally or never, had lost 6 or more teeth, were disabled, were current smokers, had self-reported "poor" or "very poor" health, or had moderate or more severe depressive symptoms.

In age-sex adjusted logistic regression models, the OR for having a dental visit among patients with dental insurance was 2.66 (95% confidence interval [CI], 2.33-3.03) compared with those without dental insurance. We found a significant interaction only between dental insurance and race/ethnicity (*P* < .001) and thus present the fully adjusted models stratified by dental insurance; these models used age, sex, education, income, and self-reported race/ethnicity (Table 2).

Among patients with dental insurance, women were more likely than men to have an annual dental visit. Compared with whites, African Americans were less likely to have an annual visit and Chinese and Filipinos were more likely. Latinos and other/mixed race were not significantly different from whites. We observed substantial differences by education and annual household income.

Among patients without dental insurance, women were more likely than men to have an annual visit, but the difference was not significant. African Americans and other/mixed race respondents were less likely than whites to have an annual visit, but Latinos, Chinese, and Filipinos were not significantly different from whites. Again, there were differences by education and annual household income.

We then calculated PPADV on the basis of fully adjusted regression models. Among people with dental insurance, although there remained differences by race/ethnicity, education, and income, the PPADV was 71% or more for all people except those with annual household incomes less than $15,000 (PPADV, 70%) (Figure). Among people without dental insurance, we found that the PPADV was 71% for college graduates, and significant only for those with annual household incomes of $65,000 or more (PPADV, 75%; 95% CI, 72%-77%). The PPADVs were 60% or less among African Americans, Latinos, Chinese, and other/mixed race, but not whites (65%). PPADVs were also less than 60% for those who did not graduate college and those with annual household incomes of less than $35,000.

**Figure.**
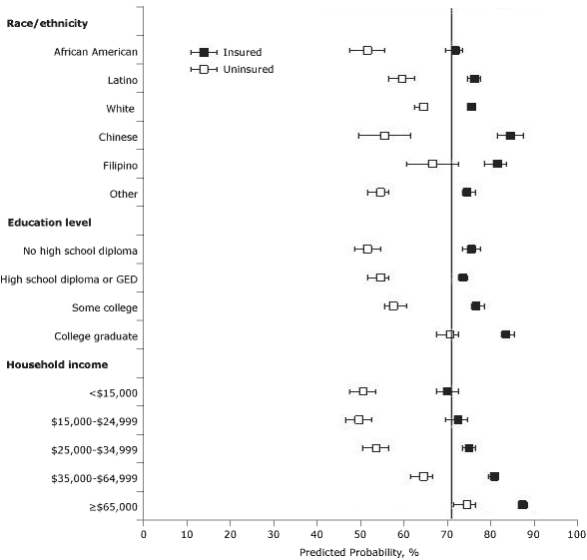
Predicted probabilities for an annual dental visit from logistic regression model, stratified by having dental insurance, using age, sex, annual income, education level, and race/ethnicity (model takes into account nonproportional sampling fractions due to oversampling by race). Error bars represent 95% confidence intervals. A subgroup meets the *Healthy People 2010* goal if prevalence of annual dental visit is 71% or more (solid line). Abbreviation: GED, General Educational Development certificate.

**Table:** 

**Characteristic**	**Insured, % (95% Confidence Interval)**	**Uninsured, % (95% Confidence Interval)**
**Race/ethnicity**		
African American	72 (70-74)	52 (48-56)
Latino	77 (75-78)	60 (57-63)
White	76 (75-77)	65 (63-66)
Chinese	85 (82-88)	56 (50-62)
Filipino	82 (79-84)	67 (61-73)
Other	75 (74-77)	55 (52-57)
**Education level**		
No high school diploma	76 (74-77)	52 (49-55)
High school diploma or GED	74 (73-75)	55 (52-57)
Some college	77 (76-79)	58 (56-61)
College graduate	84 (83-86)	71 (68-73)
**Household income**		
<$15,000	70 (68-73)	51 (48-54)
$15,000-$24,999	73 (70-75)	50 (47-53)
$25,000-$34,999	75 (74-77)	54 (51-57)
$35,000-$64,999	81 (80-82)	65 (62-67)
≥$65,000	88 (87-89)	75 (72-77)

We also observed differences by race/ethnicity in the use of dental insurance. For example, Chinese and African American patients without dental insurance had similar PPADVs, 56% and 52%, respectively. However, Chinese patients with dental insurance had a significantly higher PPADV than African Americans with dental insurance, 85% compared with 72%, respectively (*P* < .001).

We conducted sensitivity analyses to assess the effect of excluding edentulous patients and found that each of the PPADVs was reduced by 0% to 4% when we included the edentulous patients. This is consistent with 1) the proposition that patients with no teeth are less likely to have an annual dental visit and 2) our finding that having an annual visit was related to tooth loss: unadjusted annual visit rates were 81% for patients who had lost no teeth, 77% among patients who were missing 1 to 5 teeth, and 69% among those who had lost 6 or more teeth.

## Discussion

Our study is the first to examine social disparities in dental visits among medically insured patients with diabetes while taking into account dental insurance. Our results confirm previous findings of disparities in annual dental visits by race/ethnicity, income, and education level reported among patients with dental insurance (8) and patients with diabetes (13). It is difficult to compare our findings with most other studies because of different study methods (eg, not limited to people with diabetes or not stratified by dental insurance). However, our findings should be generalizable to insured, dentate patients with diabetes in California, but may not be generalizable to other regions that may have different social norms about the value of dental care.

Periodontitis was dubbed "the sixth complication of diabetes" 15 years ago (1), and the American Diabetes Association recommends that patients receive a referral for a dental examination as part of the diabetes evaluation (24). The US Surgeon General and medical and dental experts have recommended that an oral examination be part of a general medical examination and have encouraged collaboration among medical and dental providers (7,25). Unfortunately, oral health care for people with diabetes is not typically linked in any way to medical care, despite the integral role of oral health to overall health. Although the HP 2010 goal of 71% was considered realistic on the basis of prior prevalence data among older dentate adults (10), a much higher level of preventive dental care, closer to 100%, is desirable if oral health is to be maximized for patients with diabetes.

The prevalence of dental insurance among medically insured people in the San Francisco Bay area counties was 79% in the California Health Interview Survey 2003 (CHIS 2003) (9). In our study sample, the overall rate was 75% (with prevalence by race/ethnicity similar to CHIS 2003); after standardizing the prevalence to the population used by CHIS 2003, the overall rate was 72%. These rates are higher than national prevalence estimates of 2 in 3 (7).

Although African Americans and Latinos are often more likely than whites to lack medical insurance, both CHIS 2003 and our study found that, among medically insured populations, whites were the least likely racial/ethnic group to have dental insurance; this may be due to confounding by type of employment or retirement status. Data from CHIS 2003 show that people without medical insurance rarely have dental insurance and are at risk of both poor medical and poor dental care. In California, Medicaid coverage for preventive dental care was eliminated in 2009; thus, many low-income adults have lost their dental insurance, and the proportion not having an annual dental visit is likely to increase.

The large sample size and multiethnic cohort are a major strength of this study. However, because analyses were cross-sectional, we cannot make any causal inferences. The HP 2010 goal of an annual dental exam is not strictly the same as our question, which asked about recent history of "seeing a dentist for routine checkups or cleanings," but the potential for misclassification is probably small and would not have a substantive effect on findings. It is possible, though probably rare, for a patient to have dental insurance that does not cover even 1 preventive care visit, as we assumed.

As with all surveys, not all questions were answered, and thus, there is some degree of missing data. Dental insurance data were missing for 6,164 dentate people, but 94% of this was due to incomplete interviews or use of the short version of the survey, which did not contain the question. Thus, only 6% of the data were selectively missing because of respondent refusal. Finally, we lack information about patient preferences or beliefs about dental care, which might explain some of the variations in dental visits among patients with dental insurance.

In conclusion, we observed significant disparities in receipt of annual preventive dental care among medically insured patients with diabetes, often due to a lack of dental insurance, but also associated with social differences in the use of dental care or use of dental insurance. Even among those with dental insurance, social differences exist with respect to education, income, and race/ethnicity, perhaps reflecting differences in underlying attitudes toward and knowledge of the importance of dental care or of the costs and benefits of maintaining teeth. Ideally, dental and medical care will become more integrated in future health care systems. However, given the present separation of medical and dental care, health plans and diabetes health education programs should consider reviewing their approach to promoting preventive dental care as an integral and vital part of self-care, with special attention to financial barriers, cultural sensitivity, translation services, and accessibility for those with inadequate health literacy. Qualitative research approaches (eg, focus groups) may be useful for further identifying factors that may impede the use of preventive dental care. Future research is also needed to study the effect of delivery models that integrate dental and medical care on social disparities in health, particularly for high-risk patients such as those with diabetes.

## Figures and Tables

**Table 1:** Characteristics by Dental Visit Frequency of Medically Insured Patients With Diabetes in the Diabetes Study of Northern California (DISTANCE) Cohort^a^

**Characteristics**	All Subjects, n (%)	Annual Dental Visit, n (row %)	Less Than Annual Dental Visit, n (row %)
**N (%), *P* < .001**	12,405 (100)	9,492 (77)	2,913 (23)
**Dental insurance, *P* < .001**			
Yes	9,257 (75)	7,557 (82)	1,700 (18)
No	3,148 (25)	1,935 (61)	1,213 (39)
**Dental checkup frequency in past 3 years, *P* < .001**			
Twice a year	7,251 (58)	7,251 (100)	0
Once a year	2,241 (18)	2,241 (100)	0
Less than once a year	605 (5)	0	605 (100)
Whenever needed	1,742 (14)	0	1,742 (100)
Never	566 (5)	0	566 (100)
**Floss frequency, *P* < .001**			
Daily	4,997 (40)	4,156 (83)	841 (17)
Several times/week	2,475 (20)	2,050 (83)	425 (17)
At least once/week	1,127 (9)	866 (77)	261 (23)
Occasionally	2,468 (20)	1,722 (70)	746 (30)
Never	1,289 (10)	669 (52)	620 (48)
**Tooth loss, *P* < .001**			
6 or more but not all	2,750 (23)	1,904 (69)	846 (31)
1-5 teeth	4,887 (42)	3,770 (77)	1,117 (23)
No teeth lost	4,110 (35)	3,338 (81)	772 (19)
**Age, y, *P* = .007**			
30-49	2,619 (21)	1,989 (76)	630 (24)
50-59	4,197 (34)	3,244 (77)	953 (23)
60-69	3,773 (30)	2,922 (77)	851 (23)
70-75	1,816 (15)	1,337 (74)	479 (26)
**Sex, *P* = .49**			
Women	5,934 (48)	4,557 (77)	1,377 (23)
Men	6,471 (52)	4,935 (76)	1,536 (24)
**Race/ethnicity, *P* < .001**			
African American	2,064 (17)	1,501 (73)	563 (27)
Latino	2,321 (19)	1,686 (73)	635 (27)
White	3,227 (26)	2,481 (77)	746 (23)
Chinese	911 (7)	759 (83)	152 (17)
Filipino	1,294 (10)	1,078 (83)	216 (17)
Other/mixed race	2,588 (21)	1,987 (77)	601 (23)
**Employment status, *P* < .001**			
Employed	6,763 (55)	5,344 (79)	1,419 (21)
Retired	3,874 (32)	2,952 (76)	922 (24)
Disabled	874 (7)	545 (62)	329 (38)
Other (eg, student, homemaker)	711 (6)	522 (73)	189 (27)
**Education, *P* < .001**			
No high school diploma	1,607 (13)	1,065 (66)	542 (34)
High school graduate/GED	3,441 (28)	2,481 (72)	960 (28)
Some college	3,171 (26)	2,391 (75)	780 (25)
College graduate	3,994 (33)	3,419 (86)	575 (14)
**Annual household income, *P* < .001**			
<$15,000	887 (8)	527 (59)	360 (41)
$15,000-$24,999	858 (8)	548 (64)	310 (36)
$25,000-$34,999	1,267 (11)	847 (67)	420 (33)
$35,000-$64,999	3,468 (31)	2,608 (75)	860 (25)
≥$65,000 or more	4,719 (42)	4,024 (85)	695 (15)
**Smoking, *P* < .001**			
Never	7,064 (57)	5,529 (78)	1,535 (22)
Current	964 (8)	658 (68)	306 (32)
Former	4,365 (35)	3,295 (75)	1,070 (25)
**Diabetes medication, *P* < .001**			
Insulin	2,683 (22)	1,973 (74)	710 (26)
Oral agents only	8,074 (65)	6,220 (77)	1,854 (23)
No medication	1,586 (13)	1,251 (79)	335 (21)
**Self-rated general health^b^, *P* < .001**			
Excellent	2,796 (23)	2,277 (81)	519 (19)
Very good	717 (6)	603 (84)	114 (16)
Good	4,707 (38)	3,679 (78)	1,028 (22)
Fair	3,116 (25)	2,219 (71)	897 (29)
Poor	811 (7)	546 (67)	265 (33)
Very poor	213 (2)	137 (64)	76 (36)
**Depressive symptoms^c^, *P* < .001**			
Yes	1,426 (13)	934 (65)	492 (35)
No	9,647 (87)	7,568 (78)	2,079 (22)
**Body mass index, kg/m^2^, *P* < .001**			
≤30	6,133 (53)	4,835 (79)	1,298 (21)
>30	5,504 (47)	4,113 (75)	1,391 (25)
**Duration of diabetes, y, *P* = .015**			
<10	7,165 (60)	5,545 (77)	1,620 (23)
≥10	4,698 (40)	3,545 (75)	1,153 (25)
**Hemoglobin A1c, *P* < .001**			
≤8%	8,544 (76)	6,663 (78)	1,881 (22)
>8%	2,697 (24)	1,981 (73)	716 (27)

Abbreviation: GED, General Educational Development certificate.

a Numbers may not equal total because of missing data; percentages may not total 100 because of rounding. χ^2^ test for all *P* values.

b Based on SF-8 question 1 (21).

c Moderate or more severe depression, based on the 8-item Patient Health Questionnaire (PHQ8) score of 10 or higher (22,23).

**Table 2:** Odds Ratios (ORs) for Having a Dental Visit Among Medically Insured Patients, Diabetes Study of Northern California (DISTANCE) Survey Cohort, 2005-2006^a^

**Characteristic**	Insured, OR (95% CI)	Uninsured, OR (95% CI)
**Female (reference: male)**	1.38 (1.17-1.62)	1.24 (0.98-1.58)
**Race/ethnicity (reference: white)**		
African American	0.81 (0.68-0.97)	0.60 (0.45-0.80)
Latino	1.02 (0.83-1.26)	0.84 (0.63-1.11)
Chinese	1.79 (1.28-2.49)	0.69 (0.48-1.00)
Filipino	1.40 (1.09-1.79)	1.14 (0.76-1.71)
Other/mixed	0.94 (0.76-1.18)	0.67 (0.50-0.90)
**Education (reference: college graduate)**		
No high school diploma	0.58 (0.44-0.78)	0.45 (0.31-0.65)
High school graduate/GED	0.52 (0.42-0.65)	0.50 (0.36-0.70)
Some college	0.63 (0.50-0.79)	0.58 (0.41-0.82)
**Annual household income (reference: >$65,000)**		
<$15,000	0.33 (0.24-0.46)	0.36 (0.24-0.54)
$15,000-$24,999	0.37 (0.27-0.52)	0.34 (0.22-0.50)
$25,000-$34,999	0.43 (0.33-0.56)	0.40 (0.27-0.58)
$35,000-$64,999	0.60 (0.50-0.73)	0.62 (0.45-0.86)

Abbreviations: CI, confidence interval; GED, General Educational Development certificate.

a Models included age, sex, race/ethnicity, education level, and annual income; models take into account nonproportional sampling fractions due to oversampling by race/ethnicity.

## Appendix. Survey Questions About Oral Health, Diabetes Study of Northern California (DISTANCE) Survey

**Dental insurance**

Q: Do you currently have dental insurance?

□ Yes
□ No
□ Don't know

**Dental visit frequency**

Q: During the past 3 years, how often have you gone to the dentist for routine check-ups or cleanings?

□ 2 or more times a year
□ Once a year
□ Less than once a year
□ Whenever needed, no regular schedule
□ Did not go to the dentist in past 3 years
□ I wear full dentures
□ Don't know

**Flossing frequency**

Q: How often do you floss your teeth?

□ Daily
□ Several times a week
□ At least once a week
□ Occasionally
□ Never
□ Don't know
□ I wear full dentures
□ Refuse

**Tooth loss**

Q: How many of your permanent teeth have been removed because of tooth decay or gum disease? Do not include baby teeth or teeth lost for other reasons, such as injury or orthodontics. [If wisdom teeth are removed because of tooth decay or gum disease, they should be included in the count for lost teeth. Include teeth lost due to infection.]

□ All teeth lost
□ 6 or more but not all teeth
□ 1 to 5 teeth
□ None
□ Don't know
